# A synthetic population dataset for estimating small area health and socio-economic outcomes in Great Britain

**DOI:** 10.1038/s41597-022-01124-9

**Published:** 2022-01-20

**Authors:** Guoqiang Wu, Alison Heppenstall, Petra Meier, Robin Purshouse, Nik Lomax

**Affiliations:** 1 Leeds Institute for Data Analytics and School of Geography, University of Leeds, Woodhouse Lane, Leeds, West Yorkshire LS2 9JT UK; 2 Alan Turing Institute for Data Science & AI, The British Library, London, NW1 2DB UK; 3 MRC/CSO Social and Public Health Sciences Unit, University of Glasgow, Berkeley Square, 99 Berkeley Street, Glasgow, G3 7HR UK; 4 Department of Automatic Control and Systems Engineering, University of Sheffield, Portobello Street, Sheffield, S1 3JD UK

**Keywords:** Geography, Health policy

## Abstract

In order to understand the health outcomes for distinct sub-groups of the population or across different geographies, it is advantageous to be able to build bespoke groupings from individual level data. Individuals possess distinct characteristics, exhibit distinct behaviours and accumulate their own unique history of exposure or experiences. However, in most disciplines, not least public health, there is a lack of individual level data available outside of secure settings, especially covering large portions of the population. This paper provides detail on the creation of a synthetic micro dataset for individuals in Great Britain who have detailed attributes which can be used to model a wide range of health and other outcomes. These attributes are constructed from a range of sources including the United Kingdom Census, survey and administrative datasets. It provides a rationale for the need for this synthetic population, discusses methods for creating this dataset and provides some example results of different attribute distributions for distinct sub-population groups and over different geographical areas.

## Abstract (summary)

**Table:** 

Measurement(s)	Health Disparity Populations • socio-economic outcomes
Technology Type(s)	computational modeling technique • digital curation
Factor Type(s)	geographical area
Sample Characteristic - Location	Great Britain

## Background & Summary

One of the central issues that researchers and policy makers face when modelling outcomes in a public health context is access to spatially representative individual-level data. Access to this data would enable researchers to examine bespoke spatial and sub-group effects of interventions and policy scenarios, thereby assessing their equability and implications within a wider policy making context. However, access to such individual level data are understandably restricted, owing to their sensitive nature. This presents a major barrier to the development of models that can inform spatially relevant interventions in a timely fashion. One way of dealing with this is the creation of synthetic data that are representative of the relationships contained within the real population.

A well established method for creating such synthetic datasets is microsimulation. In brief, microsimulation uses attribute-rich individual-level sample data to estimate the characteristics of a larger population^1,2^. An extension of this approach that explicitly accounts for spatial distributions is often termed spatial microsimulation^3^. In both microsimulation and spatial microsimulation, the resulting synthetic population dataset can be used to simulate impacts of interventions or evaluation of policy changes at an individual level which can then be aggregated over population sub-groups or geographies to calculate the overall impact of the policy scenario^4^.

Typically, a synthetic population generated using microsimulation has a census or other large scale coverage survey as its backbone. Depending on the focus of the research agenda being addressed, this base population can be further enriched from other data sources. There are numerous examples of this approach being successfully applied to answer key policy questions which have a spatial dimension. These include the assessment of consumer expenditure patterns^5^, estimating local area infrastructure demand^6^ and health care planning in relation to the spatial distribution of morbidities^7^.

Normally, the micro component of microsimulation represents units such as individuals, households or firms, which are simulated via a process of assigning attributes to those microunits from other data sources^2^. Spatial microsimulation adds geographical constraints and allows for the synthesis of individuals within defined geographical zones^8^. This combines the advantages of non-spatial attribute-rich microdata with geographically aggregated data to synthesise a population of individuals containing characteristics from both sources. It has been widely applied in many fields such as population projections (e.g.^9,10^), health studies (e.g.^11,12^), transport analysis (e.g.^13,14^), policy evaluation (e.g.^15,16^) and assessment of deprivation and inequality (e.g.^17,18^).

In practice, spatial microsimulation models can be either *static* or *dynamic*. Whilst a static microsimulation provides a way of generating an estimated population of individuals by synthesising data, a dynamic microsimulation is able to model changes of individual units over time and ‘age’ the static population^2^. Synthetic population data have been used as an input for dynamic microsimulation^19^ and agent based models^20^. They would also lend themselves to analysis using Bayesian simulations^21^.

In this paper, we present the rationale for, and microsimulation methods used to construct a synthetic population used by the SIPHER (*Systems Science in Public Health and Health Economics Research*) consortium, a collaboration of researchers from seven universities, three government partners and 12 practice partners. SIPHER’s vision is a shift from health policy to healthy public policy^22^ One focus area for SIPHER is to understand whether a move to an inclusive economy would benefit health and social outcomes and reduce inequalities, and if so what kinds of strategic actions decision-makers could consider. Data produced in this paper will be used as an input to models geared towards assessing relationships within a systems map of an inclusive economy and the impacts of policy interventions on a range of health and social outcomes.

For the synthetic population presented in this paper, the SIPHER requirements are (i) the creation of an individual-level population at a fine geographical level for Great Britain (GB); (ii) flexibility to combine the synthetic population with data from other sources; (iii) ability to assess the distributional effects, co-benefits and trade-offs which arise from hypothetical policy interventions; and (iv) ensuring the synthetic data could be used as an input to other dynamic policy models. In this paper we present details of the construction and validation of the synthetic population for GB, and show the population synthesis results for several geographical areas as an example of data: the city region of Greater Manchester (comprising 10 local authority districts), Sheffield local authority district, Glasgow council area and Cardiff local authority district. More specifically, we demonstrate how the SIPHER baseline population is created from the 2011 UK Census, mid-year population estimates, and the Understanding Society survey dataset.

There are many ways to use these synthetic data. In their own right they can be aggregated (to create area level information not available from the original data), cross-tabulated to reveal relationships at a given spatial scale, used to calculate summaries or metrics which provide insight in to the attributes or behaviours of different groups, and “augmented” where additional data sets are attached or integrated. By experimenting on the individual data we can create scenarios, which change the distribution of attributes. These data are also useful as the input to other individual level models. They can be used in dynamic microsimulation models which age on the population and allow for experiments and scenarios to be run which incorporate time. Synthetic data can also be fed in to agent-based models which allow users to experiment with system rules and interactions between the micro-units within the synthetic population.

In the future, using our model, it will be possible to update these data to align with the results of the 2021 UK Census once they are released. Our framework can also be used to create additional microdata using other survey or administrative datasets which contain individual level information.

## Methods

**Fig. 1:**
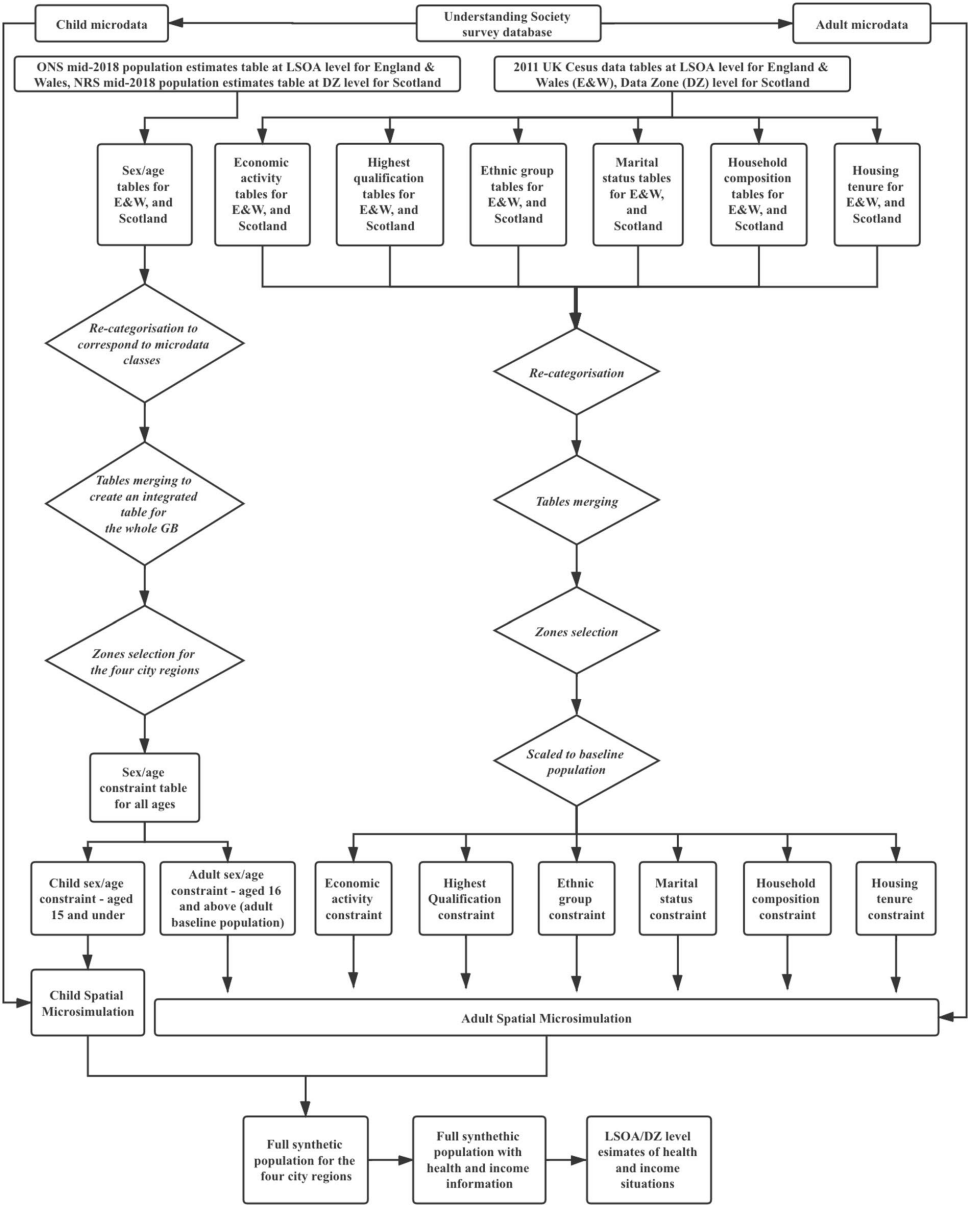
Methodological framework of generating synthetic population.

This research utilises static spatial microsimulation to produce synthetic population of individuals covering the whole of Great Britain (GB) at Lower Super Output Area (LSOA) scale (administrative areas of 1500 people) for England and Wales and Data Zones (500–1000 people) for Scotland, for the year 2018 (our base year). For these models, input data normally consists of a non-geographical but otherwise attribute-rich individual level dataset, for example a representative survey, and constraint tables containing aggregate counts across a number of attributes for a series of geographical zones (e.g. LSOAs). To calculate the weights allocated to the individuals for each geographical zone, linking variables, which are shared between the individual and aggregate level datasets, are required for setting a spatial microsimulation model^8^. In this study, two spatial microsimulation (sub-)models are created and run separately for adults (those aged 16 and over) and children (those aged 15 and under). The overall framework and procedures for generating synthetic population and health estimates in this study is shown in Fig. 1. As the source of individual level input data, the Understanding Society survey database is processed to create adult microdata for adult microsimulation model and child microdata for the child model. Corresponding to the linking variables selected and formatted in the two micro-datasets, different geographical constraint tables are formatted based on the source data from the Office for National Statistics (ONS) or National Records of Scotland (NRS) mid-2018 population estimates, and the 2011 UK Census. Both the individual level microdata and constraint data tables are fed into each of the two microsimulation (sub-)models. After each model is run, their population outputs are merged together to generate the full synthetic population and estimates of health situations at the LSOA level are created. The whole process of synthetic data generation is detailed as follows.

### Algorithm

A choice of deterministic reweighting and probabilistic methods exist for the allocation of individuals to spatial zones using microsimulation^8,23,24^. In this paper we utilise a combinatorial optimisation algorithm called simulated annealing to produce our synthetic dataset. Simulated annealing was compared to other microsimulation approaches by Harland *et al*.^1^ and found to outperform the alternatives in terms of total absolute error when comparing the microsimulation output with observed joint distributions for geographical zones.

Simulated annealing selects an optimal configuration from a small sample population (e.g. survey data) constrained by observed aggregate population counts (e.g. population census). It proceeds by randomly selecting individuals from the microdata and considering them for admittance into the population of a small area if they improve the goodness of fit of the population to the benchmark tables (constraint tables)^1,25^. The aggregation and fit evaluation is repeated, and new individuals replace the old ones in the small area if the fit is improved. Since the weights applied to members of a sample population are either one (if the individual is selected) or zero (if the individual is excluded), the synthetic population generated is a realistic representation of the observed population aligning closely to the constraint tables whilst maintaining the rich attributes provided by the survey sample population^26^.

### Software

There are a variety of ways to build and run microsimulation models. These include using custom software packages and specifying models in a development language. This study applies a Java based application, the Flexible Modelling Framework (FMF) which incorporates a static spatial microsimulation algorithm based on Simulated Annealing^27^. It also has a Graphical User Interface (GUI) which allows the users to select input data files and specify required linkages, options and outputs. In addition, the FMF includes a model evaluation function that enables internal model validation by calculating goodness-of-fit statistics (e.g. R^2^, Total Absolute Error, Standard Absolute Error, Standardised Root Mean Square Error) at individual cell, category and overall attribute levels.

### Microdata-Understanding society (the UK household longitudinal study)

The microdata (non-geographical individual level data) used for this study are derived from a nationally representative longitudinal household survey-Understanding Society (the UK Household Longitudinal Study)^?^. The initial household sample size of the first wave (2009-10) was around 40,000, and it collects data from household members aged 10 and above on an annual basis. Sample members are followed when they leave a household, and new individuals join the study as they become part of existing sample member households. The survey fieldwork period is 24 months for each wave, with each individual interviewed at 12-month intervals. The main survey of Understanding Society consists of two components, which include an individual adult survey completed by respondents aged 16 and over and a youth survey completed by young people aged 10-15. We use the adult survey data to create the input microdata for microsimulation process. Base data simulated in this paper uses wave 9 of the survey, conducted in 2018.

### Selecting and formatting linking variables in the microdata

Understanding Society’s adult survey collects a range of health and socio-economic information about individuals and a number of these align with the mid-year population estimates data and census data used as the spatial constraints (i.e. geographically aggregated dataset) in our spatial microsimulation. We use eight variables in the adult individual dataset linking to constraints: sex, age, economic activity, highest educational qualification, marital status, ethnic group, composition of household, and housing tenure. Sex and age are combined. Because unified variable classes for the microdata and corresponding constraint dataset are required for the microsimulation process, the original variables in the microdata needs to be re-categorised to generate the appropriate classes which is compatible with constraint variables derived from the mid-year population estimates and census data. These are summarised in Table 1.

**Table 1:** Summary of linking variables in Understanding Society adult microdata.

Variable	Description	Values and categories
PID	Personal identifier code	e.g. 476867687, 483520936, etc.
Sex/age	Sex and age group	M_16_24 (Male, aged 16–24 years)
		M_25_34 (Male, aged 25–34 years)
		M_35_49 (Male, aged 35–49 years)
		M_50_64 (Male, aged 50–64 years)
		M_65_74 (Male, aged 65–74 years)
		M_75+ (Male, aged 75 years and over)
		F_16_24 (Female, aged 16–24 years)
		F_25_34 (Female, aged 25–34 years)
		F_35_49 (Female, aged 35–49 years)
		F_50_64 (Female, aged 50–64 years)
		F_65_74 (Female, aged 65–74 years)
		F_75+ (Female, aged 75 years and over)
Ecostatus	Economic status	In paid employment
		Self-employed
		Unemployed
		Full-time student
		Retired
		Looking after home or family
		Long-term sick or disabled
		Others
Hiqualif	Highest educational qualification	None (no qualification)
		Level 1 or 2 (O Levels/CSE/GCSEs or equivalent)
		Level 3 (A Levels or equivalent)
		Level 4 or above (Degrees or higher degrees)
		Others (e.g. apprenticeships)
Marstat	Marital status and civil partnership status	Single
		Married
		Civil partnership
		Separated
		Divorced
		Widowed or surviving partner
Ethnicity	Ethnic group	White
		Asian
		Black
		Mixed
		Others
Hhtype	Composition of household	1_adult_no_child (1 adult only)
		1_adult_child (1 adult with child/children)
		1_couple_no_child (1 couple without child)
		1_couple_child (1 couple with child/children)
		Others_no_child (Other compositions without child)
		Others_child (Other compositions with child/children)
Tenure	Housing tenure	Owned outright
		Owned mortgage
		Social rented
		Private rented
		Others

Table 2 shows an example extract of the formatted socio-demographic microdata of adults (note these are not real records) from Understanding Society.

**Table 2:** Example of formatted adult microdata.

PID	Sex/age	Ecostatus	Hiqualif	Marstat	Ethnicity	Hhtype	Tenure
476867699	F_16_24	In_paid_employment	Level_3	Single	White	Others_no_child	Private_rented
477211091	M_75+	Retired	Others	Married	White	1_couple_no_child	Owned_outright
477285207	F_35_49	Self_employed	Level_4_above	Single	White	1_adult_child	Owned_outright
478511255	M_16_24	Student	Level_3	Single	Mixed	Others_no_child	Owned_outright
477034289	F_25_34	In_paid_employment	Others	Single	White	Others_no_child	Owned_outright
478631609	M_16_24	In_paid_employment	Level_3	Single	White	Others_no_child	Owned_outright
478913817	F_16_24	In_paid_employment	Level_3	Single	White	Others_no_child	Owned_outright

### Child microdata

The child microdata is newly-formed by extracting the information about the adult respondents’ children (aged 15 and under) recorded in the Understanding Society’s adult survey dataset. Compared to the adult microsimulation model which uses eight variables which map to the 2011 Census data, the child microsimulation model is simpler because the linking variables shared between the Understanding Society survey data and geographically aggregated data are limited. Many of the socio-demographic variables, such as economic activity, highest qualification, and marital status, are not available or applicable for children in both source datasets. A simpler microsimulation model is also deemed appropriate since the information on children’s health provided by the original child microdata is not as rich as the health-related information for adults. In this case, only sex and age variables are selected from the original child dataset to form the sex/age variable with six cross-tabulated categories for microsimulation purpose, including “M_0_4” (male, aged 0–4 years), “M_5_11” (male, aged 5–11 years), “M_12_15” (male, aged 12–15 years), “F_0_4” (female, aged 0–4 years), “F_5_11” (female, aged 5–11 years), and “F_12_ 15” (female, aged 12–15 years).

### Formatting geographical constraint variables

Tables which report the total count of individuals for each of the nine variables outlined in Table 1 for each geographical zone are used as constraints in the microsimulation. The source of these constraints are outlined in Table 3.

**Table 3:** Constraints and source datasets for adult and child microsimulation models.

Model	Constraint	Source datasets
Adult & Child	Sex/age	ONS Mid-year (2018) population estimates - 2011 LSOA based by single year of age (http://www.nomisweb.co.uk/datasets/pestsyoaoa)
		NRS Mid-2018 Small Area Population Estimates Scotland for 2011 Data Zones - by single year of age (http://www.nrscotland.gov.uk/statistics-and-data)
Adult	Economic status	2011 Census Table LC6107EW - Economic activity by sex by age for 2011 LSOA (http://www.nomisweb.co.uk/census/2011)
		2011 Scotland’s Census Table LC6107SC - Economic activity by age for 2011 Data Zone (http://www.scotlandscensus.gov.uk/ods-web/home.html)
Adult	Highest level of qualification	2011 Census Table QS501EW - Highest level of qualification for 2011 LSOA (http://www.nomisweb.co.uk/census/2011)
		2011 Scotland’s Census Table QS501SC - Highest level of qualification for 2011 Data Zone (http://www.scotlandscensus.gov.uk/ods-web/home.html)
Adult	Marital status	2011 Census Table KS103EW - Marital and civil partnership status for 2011 LSOA (http://www.nomisweb.co.uk/census/2011)
		2011 Scotland’s Census Table KS103SC - Marital and civil partnership status for 2011 Data Zone (http://www.scotlandscensus.gov.uk/ods-web/home.html)
Adult	Ethnicity	2011 Census Table LC2109EWLS - Ethnic group by age for LSOA (http://www.nomisweb.co.uk/census/2011)
		2011 Scotland’s Census Table LC2101SC - Ethnic group by age for 2011 Data Zone (http://www.scotlandscensus.gov.uk/ods-web/home.html)
Adult	Household composition	2011 Census Table LC1109EW - Household composition by age by sex for 2011 LSOA (http://www.nomisweb.co.uk/census/2011)
		2011 Scotland’s Census Table LC1109SC – Household composition by age for 2011 Data Zone (http:/www.scotlandscensus.gov.uk/ods-web/home.html)
Adult	Housing Tenure	2011 Census Table LC3409EW - General health by tenure by age for 2011 LSOA (http://www.nomisweb.co.uk/census/2011)
		2011 Scotland’s Census Table LC4302SC - Tenure by general health by long-term health problem or disability by age for Data Zone (http://www.scotlandscensus.gov.uk)

(Note: For adult model, constraints contain samples of usual residents aged 16+ derived from the original datasets. For child model, the constraint contains the derived samples of usual residents aged 15 and under).

The sex/age constraints come from the Office for National Statistics (ONS) mid-2018 population estimates for England and Wales and the National Records of Scotland (NRS) mid-2018 population estimates for Scotland. These are formatted to match the age categories in the microdata and an extract from the constraint data can be seen in Table 4. This dataset is then split in to an adult constraint dataset and a child constraint dataset. The child microsimulation model is then run using only age and sex constraints as discussed earlier.

**Table 4:** Extracted sample of the sex/age constraint for adult model.

LSOA code	M_16_24	M_25_34	M_35_49	M_50_64	M_65_74	M_75+	….	F_75+	Total (adult) baseline
E01004766	99	114	153	157	99	76	….	99	1356
E01004767	114	158	191	155	70	71	….	100	1513
E01004768	73	75	120	201	93	53	….	57	1267

(Note: Table is only for illustrative purposes and therefore does not present all categories).

For the adult model, further constraints are derived from 2011 Census data. Because the total number of people in 2011 does not match the total in 2018, the Census tables are scaled to match the age and sex totals reported in the mid-2018 data for each geographical zone. An example of the economic status and educational level constraint can be seen in Table 5, an extract from the marital status and ethnicity constraints in Table 6 and from the household composition and tenure constraint tables in Table 7. The simulated annealing algorithm is applied to these constraints and the microdata as described earlier.

**Table 5:** Extracts from the economic status and highest level of qualification constraints.

LSOA code	Economic status					Highest level of qualification			
	In paid employement	Self-employed	Retired	Full-time student	….	Level 1/2	Level 3	Level 4/above	….
E01004766	630	89	181	88	….	407	163	267	….
E01004767	697	104	183	100	….	442	212	312	….
E01004768	693	148	184	84	….	379	169	488	….

(Note: Table is only for illustrative purposes and therefore does not present all categories).

**Table 6:** Extracts from the marital status and ethnicity constraints.

LSOA code	Marital status					Ethnicity				
	Single	Married	Separated	Divorced	….	White	Mixed	Asian	Black	Others
E01004766	464	502	51	156	….	1375	26	161	28	2
E01004767	511	594	54	120	….	1337	30	298	10	3
E01004768	279	826	24	88	….	1501	28	63	4	5

(Note: Table is only for illustrative purposes and therefore does not present all categories).

**Table 7:** Extracts from the household composition and housing tenure constraints.

LSOA code	Household composition					Housing tenure			
	1 adult no child	1adult with child	1 couple no child	1 couple with child	….	Owned outright	Owned mortgage	Social rented	….
E01004766	350	222	340	544	….	440	636	180	….
E01004767	286	143	398	646	….	527	694	85	….
E01004768	100	90	422	872	….	519	970	16	….

(Note: Table is only for illustrative purposes and therefore does not present all categories).

## Usage Notes

Once the microsimulation process is complete, the synthetic population data, containing individual personal identifiers and the codes of LSOA zones that each individual is allocated to, are generated. This means that variables that are not otherwise available at a high resolution geography can be made available to researchers. Examples of these variables are provided in Table 8, including subjective wellbeing, physical and mental health conditions, and household income, all of which are reported in original Understanding Society’s adult survey data but not readily available from any existing geographically aggregated data sources. In the original survey data, subjective wellbeing scales run from 0 to 36 for Likert score and 0 to 12 for Caseness score. For either scoring method, a higher score suggests a more distressed situation the individuals are facing. SF-12 Physical or Mental Component Summary is a continuous scale with a range of 0 (low functioning) to 100 (high functioning). A higher score indicates a healthier condition the individuals have.

**Table 8:** Example of individual health and household income summaries in adult microdata.

PID	Subjective wellbeing (Likert score)	Subjective wellbeing (Caseness score)	SF-12 Physical Component Summary	SF-12 Mental Component Summary	Total household net income per month (£)
476867699	14	4	58.69	31.54	2459.68
477211091	10	0	42.18	49.02	2068.67
477285207	17	6	28.86	37.48	2986.60
478511255	7	0	46.55	55.97	6823.43
477034289	16	5	61.80	48.18	3614.15
478631609	6	0	59.91	52.21	7750.00
478913817	18	5	52.18	25.61	2642.31

**Fig. 2:**
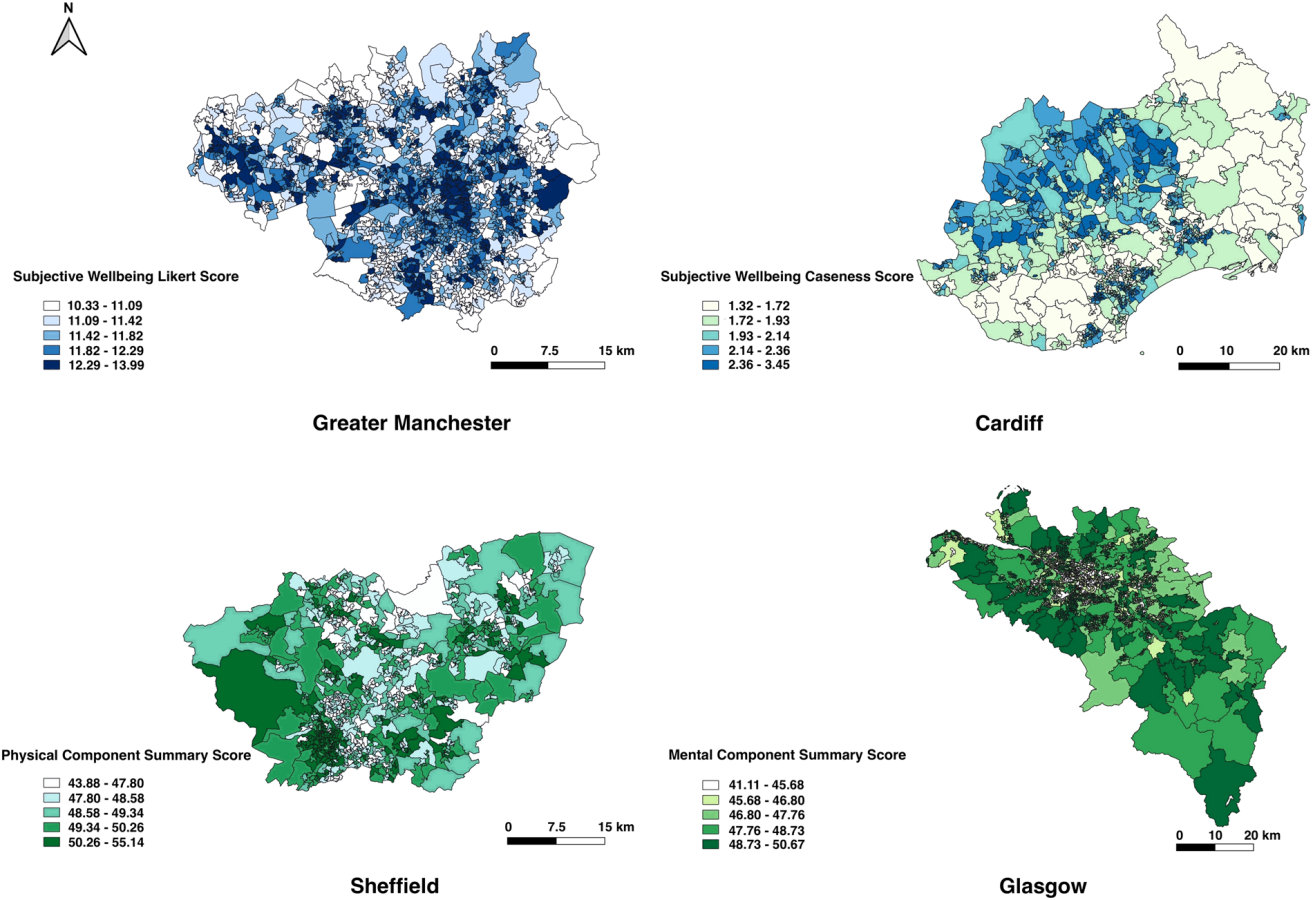
Examples of aggregated health conditions estimates at LSOA and equivalent level in the four selected city regions.

The spatial distributions of these variables (and any other variables within the dataset) can be mapped by joining the geographically aggregated dataset with the 2011 Census geography boundaries. Figure 2 gives an example of the spatial resolution of the data that is deposited across the four different GB city regions.

## Data Records

The dataset of aggregated average health conditions and average household income at the LSOA level for the four city regions described above is publicly and freely available through Figshare^28^. The input datasets for microsimulation model development, Understanding Society^29^ survey data, can be accessed through the UK Data Service. Accessing datasets from the UK Data Service normally requires online registration if users are in the UK and their organisation is part of the UK Access Management Federation (UKAMF). For users who are not in the UK or their organisation is not on the list of the UKAMF, an online application for username is required before they can register on the UK Data Service (more details are available from: https://beta.ukdataservice.ac.uk/myaccount/login). Meanwhile, the geographically aggregated data used to form constraints can be accessed from the sources listed in Table 3.

## Technical Validation

There are a range of methods available for validation of spatial microsimulation models, which can be broadly categorised as internal (in-sample, or endogenous) validation and external (out-of-sample, or exogenous) validation^23,30^.

### Internal validation

Internal validation refers to comparing values from the simulated dataset to the original datasets used in the simulation^8^. In practice, this process includes model calibration, whereby the model fit is assessed by comparing the observed and simulated values for constraint variables. Although there are a variety of established measures of internal fit that have been used for model validation, no consensus has been reached yet regarding the most meaningful measure to compare simulated and actual values^31^. Following suggestions from Lovelace and Dumont^8^, internal validation was performed by calculating the overall values of three commonly-used fit statistics: R^2^, Standardised Root Mean Square Error (SRMSE), and Relative Error (RE) for each constraint variable (summarised in Table 9). The goodness-of-fit statistics show a good fit between the simulated and original data: correlations are high and relative error is low.

**Table 9:** Validation metrics for the comparison of simulated and actual counts in each constraint.

Constraint	R^2^	SRSME	RE
Sex/age	0.999997	0.000478	0.000002
Economic status	0.999759	0.114631	0.005403
Highest level of qualification	0.986956	0.270564	0.014439
Marital status	0.999968	0.085743	0.002326
Ethnicity	0.999991	0.001056	0.000095
Household composition	0.995798	0.246496	0.009777
Housing tenure	0.984656	0.320565	0.020327

### External validation

External validation is the process of comparing the simulated results to a different source of data that is external to the model. If external geo-coded survey data are available, this approach can take place at the individual level. But more commonly, it is performed at the aggregated level^8^ given that microsimulation models are used to estimate the data that do not otherwise exist or are not accessible. In this study, the simulation results are compared with estimates from external datasets that are available at higher levels of spatial aggregation (Lower and Middle Layer Super Output Areas). Simulated outputs are aggregated up to match the geographical scale.

The Index of Multiple Deprivation 2019 (IMD 2019) (https://www.gov.uk/government/statistics/english-indices-of-deprivation-2019), the Welsh Index of Multiple Deprivation 2019 (WIMD 2019) (https://gov.wales/welsh-index-multiple-deprivation-full-index-update-ranks-2019), and the Scottish Index of Multiple Deprivation 2020 (SIMD 2020) (https://www.gov.scot/collections/scottish-index-of-multiple-deprivation-2020/) are the official measurements of relative deprivation for small areas (LSOAs or Data Zones) in England, Wales, and Scotland. Several studies have examined the relationships between socio-economic status, deprivation and individuals’ health conditions, as well as (personal or household) income. These studies generally suggest that people from more affluent households, less deprived areas or of a higher socio-economic status tend to have higher-level incomes and wellbeing and are in better health (e.g.^32–36^).

The IMD 2019 is organised across seven distinct domains of deprivation: income deprivation, employment deprivation, health deprivation and disability, education skills and training deprivation, barriers to housing and services, living environment deprivation, and crime. Those domains are combined and weighted appropriately to calculate the IMD which represents an overall measure of multiple deprivation experienced by people living in specific areas (LSOAs). All areas in England are then ranked according to their level of deprivation relative to that of other areas, and a higher rank suggests a more deprived situation. The WIMD 2019 and the SIMD 2020 are organised similarly, though some domains of deprivation are named in slightly different ways. While some of those deprivation domains are similar to the constraints used in our microsimulation model (e.g. employment deprivation, education skills and training deprivation), there are no common datasets between the IMD/WIMD/SIMD and the microsimulation. Moreover, many of the deprivation domains are not included in microsimulation (e.g. barriers to housing and services, living environment deprivation, crime). This minimises the risk of circularity when examining relationships. Spearman’s test of rank correlation was used to examine the relationships between the IMD 2019, WIMD 2019, and SIMD 2020 ranks and our simulated income and health estimates for different city regions, with the results shown in Table 10.

**Table 10:** Correlations between IMD/WIMD/SIMD rank and microsimulation estimates of income and health conditions at LSOA or Data Zone level.

	Spearman’s Rho		
	England	Wales	Scotland
	(Greater Manchester & Sheffield)	(Cardiff)	(Glasgow)
Total household net income per month	0.8718 (P-value < 0.0001)	0.8842 (P-value < 0.0001)	0.8591 (P-value < 0.0001)
SF-12 Physical Component Summary	0.7224 (P-value < 0.0001)	0.7352 (P-value < 0.0001)	0.7641 (P-value < 0.0001)
SF-12 Mental Component Summary	0.8704 (P-value < 0.0001)	0.8839 (P-value < 0.0001)	0.8454 (P-value < 0.0001)
Subjective wellbeing (Likert score)	−0.9041 (P-value < 0.0001)	−0.9174 (P-value < 0.0001)	−0.8867 (P-value < 0.0001)
Subjective wellbeing (Caseness score)	−0.8992 (P-value < 0.0001)	−0.9135 (P-value < 0.0001)	−0.8783 (P-value < 0.0001)

All correlations are significant, and the correlations between the IMD/WIMD/SIMD rank and simulated household income and (physical and mental) health summaries are positive. This suggests that individuals living in more deprived areas (with higher IMD/WIMD/SIMD ranks) tend to have lower incomes and worse health conditions; this is as expected^32,33,36^. For subjective well-being, a higher score means lower well-being. Negative correlations with IMD/WIMD/SIMD ranks suggest lower well-being in more deprived areas, which is in line with previous research (e.g.^34,37^). These results suggest that household income and health conditions at the small area level are in line with results from the independent IMD data.

**Fig. 3:**
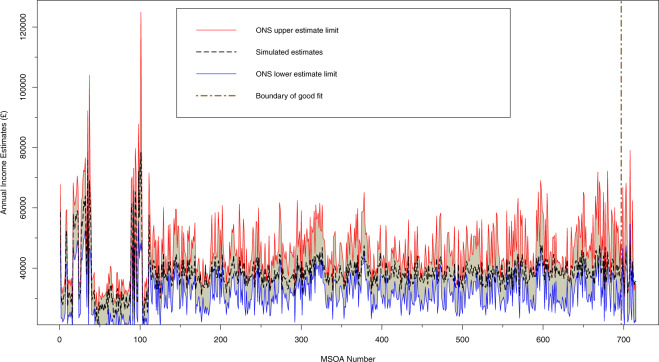
Comparison of microsimulation estimates of annual household net income and ONS income estimates at MSOA level.

In order to validate the income estimates in our synthetic data we compare results with the 2018 income estimates for small areas (IESA) (https://www.ons.gov.uk/employmentandlabourmarket/peopleinwork/earningsandworkinghours/datasets/smallareaincomeestimatesformiddlelayersuperoutputareasenglandandwales) for England and Wales published by ONS (no equivalent is available for Scotland). The 2018 IESA datasets are published at MSOA level and we aggregate the synthetic data outputs for the three English and Welsh cities (Cardiff, Greater Manchester and Sheffield) to match, resulting in 715 MSOAs. We calculate annual net income from monthly net income in our data. The IESA provide a main estimate as well as confidence intervals for each MSOA. Figure 3 provides a summary of this comparison.

Red and blue solid lines represent the upper and lower confidence limits of the ONS estimates, while the black dashed line refers to our simulated income estimates. In addition, there is a brown dashed line perpendicular to the x-axis in the diagram (i.e. the “boundary of good fit” line), separating the MSOAs (on the left side of the line) where the simulated estimates fall into the confidence intervals formed by the upper and lower limits of the ONS estimates, from those (on the right side of the line) where the simulated estimates fall out of such intervals. The microsimulation derived income estimates fall within the ONS confidence intervals in 97% of MSOAs assessed (696 out of 715). For the remaining 3% of MSOAs we examined if these areas were spatially correlated with each other, and found that they are randomly distributed within the three city regions without correlations or clustering.

In summary, both the internal and external validations results above suggest that the simulated population has captured well the differences in individuals’ health and income situations at the small area level.

## Data Availability

The Java based FMF software used to create the synthetic microdata is made available for free under a GNU General Public Licence and can be downloaded from: https://github.com/MassAtLeeds/FMF/releases. The R (version 4.0.3; https://www.r-project.org) code developed for generation, aggregation, and validation of the synthetic microdata are publicly and freely accessible through Figshare^28^. The script is documented to both explain its purpose and guide the user through its customisation.
